# Maximising Potential in Organ Donation and Transplantation: Transferrable Paradigms

**DOI:** 10.3389/ti.2022.11005

**Published:** 2023-05-25

**Authors:** Charlotte Johnston-Webber, George Wharton, Elias Mossialos, Vassilios Papalois

**Affiliations:** ^1^ Department of Health Policy, London School of Economics and Political Science, London, United Kingdom; ^2^ Institute of Global Health Innovation, Imperial College, London, United Kingdom; ^3^ Renal and Transplant Unit, Hammersmith Hospital, Imperial College Healthcare NHS Trust, London, United Kingdom; ^4^ Department of Surgery, Imperial College Renal and Transplant Unit, London, United Kingdom

**Keywords:** organ donation, policy, organ transplant, comparative analysis, health systems

Organ donation and transplantation is one of the great success stories of modern medicine, offering the chance of a new life for those with organ failure. The astonishing advancements in medical science, surgical and medical care of the past few decades have taken this treatment option from dream to reality and has transformed the lives of tens of thousands of transplant recipients and their families around the world. However, despite this progress, building an effective and efficient organ donation and transplantation program that meets the needs of a population remains a difficult task, fraught with challenges. Having medical professionals and managers in possession of the requisite knowledge and skills, as well as access to appropriate facilities, are not sufficient to achieve success in this field. Many other factors must be addressed such as gaining continual governmental commitment, investing in adequate infrastructure, implementing carefully designed reimbursement mechanisms and maintaining a highly trained and motivated workforce in sufficient numbers. Moreover, the demand for organs constantly outstrips the supply (1), highlighting the need to focus on the donation end of the process, and the need to gain the support and trust of the public (2). It also underlines the need for investment in public health and primary care programmes which have the potential to reduce the risk of organ failure and therefore reduce the need for transplant (3).

Assessing the performance of national organ donation and transplantation programs, and identifying areas in which there is need for improvement is therefore a complex task requiring attention to many different components. In order to function optimally, many healthcare processes require the collaboration and coordination of a large number of different actors. Organ donation and transplantation is a good example of a such a process which transcends many different scientific disciplines and medical specialities and which cannot function optimally without a high degree of collaboration. Assessing performance in such processes therefore requires careful and thoughtful system-wide analysis in order to gain a full understanding of the challenges and to identify areas in need for improvement. Over recent years, conceptual frameworks have been increasingly used in the assessment of healthcare systems, and they can play an invaluable role in assisting in these analyses (4–6), enabling them to be conducted in a systematic and scientifically sound manner.

In this special issue of Transplant International, we present a conceptual framework which provides a blueprint for the components of successful national organ donation and transplant programs, and can be used for their assessment. Applying this framework, we then present a series of country case studies that highlight the distinctive features that have contributed to the success of the organ donation and transplant programs of Croatia, Italy, Portugal, Spain, and the United Kingdom and elaborate on the lessons that can be learned from them. We also apply this framework to analyse the Greek system, a comparatively weaker performer, and make recommendations for its reform and development. Together, the framework and case studies demonstrate the common features of successful organ donation and transplantation systems, and highlight transferrable elements that can be applied elsewhere with the aim of improving performance.

Our case studies highlight a number of important considerations and examples of best practice, from which other countries can learn. It is clear that every organ donation and transplantation programme must be designed with the country’s particular cultural context and resource capacity in mind. Organ donation, in particular, is a highly emotive issue which inevitably raises challenging ethical questions (7). Issues such as the diagnosis of death by neurological criteria may challenge prevailing cultural or religious concepts of death and need to be carefully addressed in a transparent, empathetic and culturally sensitive manner. However, our case studies offer good examples of success, some which have achieved this despite relatively modest financial means (Mah et al., Streit et al.). Spain is widely regarded as a leader in the field (Streit et al.), with an organisational structure and strategy which has been effectively adopted by other European countries such as Portugal and Italy (Streit et al., Mah et al.). The three-tier structure of the Spanish program, with highly trained organ donation coordinators as a key component has been an extremely successful approach which many other countries have used as a blueprint for their own program. Meanwhile, in Portugal a series of reforms in the governance of dialysis units have contributed to a reduction in the out-of-pocket payments made by patients, and improvements in quality and outcomes (Streit et al.). Furthermore, in the UK, the successful integration of programmes for research and development with clinical practice have yielded significant insights into means of improving the efficiency of transplantation services (Johnston-Webber et al.), while Italy has pioneered the implementation of innovations in clinical practice and transplant related technologies as well as efficient public awareness campaigns which hold the promise of significantly expanding the available donor base (Mah et al.).

The success of these countries is underpinned by some important common elements: strong support from government; effective public awareness campaigns and relations with the media to build trust and support; an inclusive approach to policy development underpinned by public and expert consultations; and an adequately funded and staffed national transplant organisation which plays a critical role in coordinating activities across the system. As with many other areas of medicine, prevention is better than cure, and successful transplantation systems are best seen as a vital complement to effective public health programmes and universally accessible, well-integrated primary care and specialist services to detect, treat and manage the causes of organ failure, as well as social programmes which address the wider determinants of health (8).

These examples are instructive for countries whose organ donation and transplantation services are at an early stage of development or remain too fragmented to make transplantation a feasible treatment option for all but a lucky few. A case in point is Greece: our study of the Greek system highlights significant shortfalls in funding, public support, staffing, infrastructure, and operational elements necessary for a high-performing organ donation and transplantation system, which are reflected in low transplantation rates and correspondingly high levels of renal replacement therapy, driven by high incidence and prevalence of organ failure among the population. Correspondingly, we propose a set of recommendations, based on an analysis of the Greek system, the lessons learned from our case studies and consultation with a wide array of stakeholders, to enable Greece to attain rates of donation and transplantation in line with those of its European counterparts.

It is our hope that the framework and the case studies will provide a valuable template for the assessment of national organ donation and transplantation systems beyond those included in our sample, to enable the identification and prioritisation of policies to improve their efficiency, responsiveness and availability, with the ultimate goal of improving the health and care of people with organ failure.
